# Prevalence and Correlates of Physical Inactivity in Community-Dwelling Older Adults in Ireland

**DOI:** 10.1371/journal.pone.0118293

**Published:** 2015-02-11

**Authors:** Elaine M. Murtagh, Marie H. Murphy, Niamh M. Murphy, Catherine Woods, Alan M. Nevill, Aoife Lane

**Affiliations:** 1 Department of Arts Education and Physical Education, Mary Immaculate College, University of Limerick, Limerick, Ireland; 2 Sport and Exercise Science Research Institute, University of Ulster, Newtownabbey, Co. Antrim, Northern Ireland; 3 Centre for Health Behaviour Research, Waterford Institute of Technology, Waterford, Ireland; 4 School of Health and Human Performance, Dublin City University, Dublin, Ireland; 5 Faculty of Education, Health and Wellbeing, University of Wolverhampton, Walsall, United Kingdom; University of Akron, UNITED STATES

## Abstract

The public health challenges associated with rapid population ageing are likely to be exacerbated by poor physical activity levels. The purpose of this study was to identify correlates of physical inactivity in a population-representative sample of older adults in Ireland. This paper reports a secondary analysis of data from 4892 adults aged 60+ from the Irish Longitudinal Study on Ageing (TILDA). TILDA includes an assessment of the mental and physical health, and social and financial circumstances of participants assessed in a home interview and self-completion questionnaire. Chi squared statistics and forced entry logistic regression were used to identify factors associated with physical inactivity. Females were over twice as likely to be inactive as their male counterparts (Odds Ratio 2.2). Increasing old age was associated with inactivity among males and females. Those who reported above secondary level education, no reported falls in the last year and no fear of falling were less likely to be physically inactive. While older adults who noted poor/fair self-reported health, that they did not look after grandchildren, did not own a car or did not attend a course were also more likely to be inactive than those who reported positively for these items. Gender displayed a strong but often contrasting influence on factors that affect physical activity among older adults. Among females, living alone or in a rural area, retirement, fair/poor emotional health and activity being limited by illness were all significantly associated with inactivity. While cohabiting, being employed and residing in an urban area were related to low levels of activity in males. Our findings identify specific groups of the older Irish population who may be at particular risk of physical inactivity and thereby the associated physiological and psychological hazards. These results can support the development of tailored interventions to promote healthy ageing.

## Introduction

Physical inactivity is the 4^th^ leading cause of death worldwide [1]. Inactivity—that is, an activity level insufficient to meet present recommendations [2]—increases the risk of many adverse health conditions, including diseases such as cardiovascular disease, type 2 diabetes and breast and colon cancers, and shortens life expectancy [3]. Approximately one third of adults worldwide are inactive and older adults are at particular risk of inactivity [4]. Indeed, the decline in physical activity with age is one of the most consistent observations in behavioural epidemiology [5]. This can be partially attributed to the ageing process where structural and functional changes to the cardiovascular, muscular and skeletal systems impact the ability to be active [6,7]. However, being physically active throughout the lifespan can help maintain health and reduce this decline in physical function while also imparting important cognitive and psychological benefits to the ageing adult [8–11].

Ireland shares the prospect of rapid and sustained population ageing with other developed countries and this change in the demographic profile poses a major public health challenge [12]. It is predicted that the number of people aged 65 and over in Ireland will rise from 532,000 in 2011 to almost 1.4 million by 2046 [13]. It has been suggested that given the breadth and strength of the evidence, physical activity should be one of the highest priorities for preventing and treating disease and disablement in older adults [8]. The World Health Organisation recommends that older adults should engage in 150 minutes of at least moderate intensity activity per week and muscle strengthening activity twice weekly to accumulate related health benefits of being active [2]. A systematic review by Sun et al. [5] indicated that the proportion of older adults who are sufficiently active (i.e. meeting WHO guidelines) ranges between 20–50% using self-report data and 2–12% using objective data. In order to effectively develop interventions to address this widespread physical inactivity, an extensive study of the correlates of physical inactivity is required.

Socio-ecological models suggest that behaviours such as physical activity have multiple levels of influences, often including intrapersonal (biological, psychological), interpersonal (social, cultural), organisational, community, physical environmental, and policy [14]. Socio-ecological approaches also emphasise the importance of the inter-connections between individuals, their environment and the subsequent impact on behaviour [15]. This perspective therefore proposes that understanding the multiple and interacting determinants of health behaviours is essential when attempting to change behaviour [14]. Correlates of physical activity have been studied across the various levels of the ecological model and a recent comprehensive review indicated that for older adults biological (younger age, male sex), psychosocial (favourable health status, self-efficacy), demographic (higher education) and interpersonal (social support) correlates are all related to being physically active [16]. However no consistent environmental correlates were identified for older adults [16]. In an earlier review of longitudinal studies Koeneman et al. [17] reported additional biological correlates (general physical functioning and absence of disease) that were associated with exercise participation. While using the socio-ecological approach to understand the factors which influence physical activity behaviour has been undertaken internationally [18–20], to date there is no published study which has specifically examined the Irish population. The purpose of this analysis is to identify the prevalence of physical inactivity and determine individual, interpersonal and environmental correlates of this behaviour in a nationally representative sample of older adults living in Ireland.

## Methods

### Source data

This study involved an analysis of data collected as part of the Irish Longitudinal Study on Ageing (TILDA). The design and methodology of TILDA is described in detail elsewhere [21]. In brief, TILDA is a population-representative prospective cohort study of community-dwelling adults in the Republic of Ireland aged 50 and older (n = 8175). Participants were recruited from a cluster random sample of all households in Ireland [22]. Ethical approval was obtained from the Trinity College Dublin Research Ethics Committee and written informed consent was required from participants. TILDA includes detailed assessment of the mental and physical health and social and financial circumstances of participants, which were assessed in a home interview using computer-assisted personal interviewing (CAPI) and a self-completion questionnaire [21]. A detailed health assessment then took place at a dedicated health centre or in the respondent’s home. The response rate for interviews was 62%, and the response rate to the self-completion questionnaire was ~84% [12]. This paper includes data from 4892 adults aged 60+ from wave 1 of the study, which was collected between October 2009 and February 2011.

### Physical activity assessment

Physical activity was assessed using the International Physical Activity Questionnaire (IPAQ) short form, which has demonstrated acceptable reliability and validity [23]. The IPAQ scoring protocol categorises respondents as ‘low, ‘moderate’ or ‘high’ levels of physical activity [24]. Individuals who are categorised as high or moderately active technically meet minimum physical activity guidelines [25]. However, an alternative interpretation of IPAQ data has been proposed by several researchers, which suggests that only participants categorised as high active meet minimum physical activity requirements [24,26]. As IPAQ assesses multiple domains of physical activity, the level described as ‘moderate’ in the IPAQ scoring protocol would be achieved by most adults through background activity, such as work, housekeeping, and family care, that adults accumulate daily [26]. Although, high active reflects physical activity levels greater than those recommended as standard or minimum, it provides more accurate estimates of sufficient activity for participants who detail the specific nature and extent of their engagement in physical activity, as per the IPAQ instrument. The IPAQ (2005) also noted that high active is more suitable and appropriate as a unit of comparison for assessments of physical activity levels across various population sub groups. Given these observations and the limitations of using IPAQ with older adults [27], in this paper the ‘high’ category was used to deem participants as meeting the minimum physical activity guidelines of 150 minutes of moderate intensity activity weekly [28,29]. Participants were then classified as either active (i.e. meeting or exceeding guidelines) or inactive (i.e. not meeting guidelines).

### Potential correlates of physical activity

In this paper 17 independent variables were considered. These included eight demographic variables (age, SES, education, living status, children, employment status, car ownership and sex), six health-related variables (self-rated overall health, self-rated emotional health, falls in the last year, fear of falling, long term health problem, activity limited by illness), two social/cultural (looking after grandchildren, attending a course) and one environmental (geographical location). Data for attending a course were obtained during the self-completion questionnaire. Information for the other variables was obtained during the home-based interview. We used the ‘social economic group’ (category A-G) variable for this analysis. The highest social group (SC1–2) included individuals classified as A, B or C (professional and managerial), SC3–4 included category D and E (non-manual/skilled manual) and the lowest SES grouping (SC5–6) represented semi-skilled and unskilled occupations (F, G). Education was categorised as having or not having tertiary education. Living status was dichotomised into living with someone or alone. Having children and car ownership was categorised as yes or no responses. Employment status categorised respondents dichotomously as employed or retired. Questions regarding self-related overall and emotional health were scored on a scale and then dichotomised into fair/poor or good/very good/excellent. All other health-related, psychosocial and social/cultural variables were dichotomised into yes or no. Area of residence was categorised into urban or rural. The interviewer noted the location of the dwelling as being either in Dublin city or county (urban), another town or city (urban), or a rural area (rural). The exact wording of all questions used in the survey is available online [30].

### Data analysis

Participants were grouped into age categories (60–64, 65–69, 70–74 and 75+) and all subsequent analysis was undertaken on this specific group of participants. Chi squared statistics and forced entry logistic regression was used to identify factors associated with inactivity, using SPSS Version 19. Data were presented as adjusted odds ratios (OR) of the likelihood of the specified outcome (physical inactivity) across correlates which included age, gender, SES, level of education and urban/rural location. Logistic regression revealed a significant interaction between gender and several other categorical correlates so ORs are presented for males and females separately. Probability values and 95% confidence intervals for each adjusted OR are presented. ORs greater than 1 indicate an increased likelihood of being physically inactive while ORs less than 1 represent a decreased likelihood of being inactive. Weighting of variables was employed to facilitate the generalisation of findings to the overall older adult population in the Republic of Ireland. Significance was set at 0.05.

## Results

A description of participants is shown in Table 1. Females represented 53.4% of the sample. Only 2.9% of the sample was non-Irish. Eighty-five percent of participants did not have above secondary education. Table 2 shows the proportion of adults in the low, moderate and high IPAQ categories according to age. The high category equates to meeting or exceeding current physical activity guidelines. There was a significant difference in physical activity levels across all age groups (60–64, 65–69, 70–74, 75+), specifically the proportion of adults deemed active decline consistently with increasing age. Thirty eight percent of adults aged 60–64 met physical activity guidelines while only 18% of those aged 75+ did so.

**Table 1:** Description of participants aged 60+ years in TILDA (n = 4892) (weighted sample characteristics).

	%
**Sex**	
Male	46.6
Female	53.4
**Age**	
60–64	30.1
65–69	21.6
70–74	17.7
75+	30.5
**Socioeconomic status**	
SC1–2	31.9
SC3–4	39.0
SC5–6	29.1
**Education level**	
Secondary or below	85.5
Above secondary	14.5
**Ethnicity**	
Irish	97.7
Other	2.9
**Marital status/living arrangement**	
Single/Widowed/Divorced/Separated	49.1
Married/Cohabiting	50.9
**Location**	
Urban	50.7
Rural	49.3

**Table 2:** The proportion of adults classified as physically inactive / active according to age.

	Physical Activity Level	
	Inactive	Active
60–64	62.7	37.3
65–69	67.6	32.4*
70–74	72.0	28.0*
75+	82.3	17.7*

*p<0.05, Significant difference in the proportion of physically active adults across all age groups

Females were over twice as likely to be inactive than their male counterparts (OR 2.20 (95%CI 2.06–2.36). Table 3 shows the results of the logistic regression analysis with gender interactions. The interactions between gender and all other correlates with the exception of children and car ownership was significant, which suggests that gender has a strong influence on factors that affect physical activity among older adults. Increasing age was associated with inactivity among males and females while those who reported above secondary level education, no reported falls in the last year and no fear of falling were less likely to be inactive. Similarly, all older adults who perceived their overall health as fair or poor were more likely to be inactive than those who reported their health as excellent/very good/good, while adults who reported that they did not look after grandchildren, did not own a car or did not report attending a course were also more likely to be inactive than those who reported positively for these items. Among females, living alone or in a rural area, retirement, fair/poor emotional health and being limited by illness were all significantly associated with inactivity while cohabiting, being employed and residing in an urban area were related (p<.05) to low levels of activity in males.

**Table 3:** Odds Ratio (OR) of physical inactivity by demographic, health-related and environmental variables with gender interactions.

Variable		Adjusted OR (95% CI)	
		Female	Male
Constant		1.24	2.74
**Demographic:**			
Age	60–64	1.00	1.00
	65–69	1.19 (1.15–1.24) ^	1.02 (.97–1.06)
	70–74	1.27 (1.22–1.32) ^	1.56 (1.48–2.40) ^
	75+	2.13 (2.05–2.23) ^	2.53 (2.40–2.66) ^
SES	High	1.00	1.00
	Middle	0.94 (0.91–0.97) ^	0.81 (0.78–0.85) ^
	Low	0.91 (0.88–0.94) ^	1.02 (0.98–1.07)
Education	No third Level	1.00	1.00
	Third Level	0.79 (0.76–0.82) ^	0.89 (0.86–0.93) ^
Living Status	Living w/someone	1.00	1.00
	Living alone	1.14 (1.11–1.18) ^	0.72 (0.69–0.74) ^
Children	Yes	1.00	1.00
	No	0.96 (0.93–1.00)	0.95 (0.91–0.99) ^
Employment Status	Employed	1.00	1.00
	Retired	1.48 (1.42–1.54) ^	0.82 (0.79–0.86) ^
Car Ownership	Yes	1.00	1.00
	No	1.35 (1.30–1.41) ^	1.36 (1.31–1.42) ^
**Health-related:**			
Perceived Overall Health	Excellent/Very good/Good	1.00	1.00
	Fair/Poor	1.46 (1.38–1.54) ^	1.15 (1.09–1.21) ^
Perceived Emotional Health	Excellent/Very good/Good	1.00	1.00
	Fair/Poor	2.60 (2.38–2.84) ^	0.95 (0.89–1.02)
Falls in Last Year	Yes	1.00	1.00
	No	0.88 (0.83–0.94) ^	0.45 (0.42–0.48) ^
Fear of Falling	Yes	1.00	1.00
	No	0.47 (0.44–0.50) ^	0.52 (0.49–0.55) ^
Activity limited by illness	Yes	1.00	1.00
	No	0.73 (0.69–0.77) ^	0.95 (0.91–1.00)
**Social/Cultural:**			
Look after grandchildren	Yes	1.00	1.00
	No	1.15 (1.09–1.21) ^	2.30 (2.18–2.42) ^
Attend a Course	Yes	1.00	1.00
	No	1.15 (1.03–1.28) ^	1.18 (1.09–1.27) ^
**Environmental:**			
Location	Rural	1.00	1.00
	Urban	0.87 (0.84–0.89) ^	1.24 (1.20–1.28)^

^OR significant (p<.05)

## Discussion

This is the first paper to report on correlates of physical inactivity in a population-representative sample of older adults in the Republic of Ireland. Our findings identify specific groups of the Irish older adult population who are at particular risk of physical inactivity and thereby the associated physiological and psychological hazards of an inactive lifestyle. Consistent with previous research [16], gender emerged as a strong correlate of physical activity in the current study. Females were 2.2 times more likely than males to be inactive. Multiple reasons like family and societal roles, psychological issues, and life conditions may account for these differences [31]. A recent study in German older adults indicated that older men engaged in sporting activities more often than women, while women performed more domestic activities [32]. Older adults may therefore experience more conventional role assignment than is currently the case among younger adults, and while the domestic activities undertaken by women may compensate for low participation in sporting activity, it may not yield the same benefits for social and psychological health [33]. It has been suggested that the distinct intrapersonal characteristics that influence women’s physical activity, such as motivation and affective health, need to be identified before interventions can be developed to meet their unique needs [34]. There is some evidence to suggest that once involved in a regular programme of physical activity adherence rates may be similar for both sexes, highlighting that physical activity promotion in earlier life may be essential for ensuring sufficient levels among older women [35].

The present study revealed unique correlates of inactivity for men and women. Among females, living alone or being retired were significantly associated with increased odds of inactivity, while the opposite relationship was evident for men, i.e. those who lived alone or were retired were less likely to be physically inactive. It has been noted in a New Zealand study that women without partners were particularly disadvantaged in terms of their living standards and financial preparations during retirement[36], which may have an impact on access to physical activity participation. Low self-rated emotional health was strongly associated with inactivity for females (OR 2.6) but not males. It has been proposed that women’s self-assessed health judgments are based on a wider range of health-related and non–health-related factors than are men’s, which can explain gender differences and may be related to other documented differences in women’s mental health and illness behavior [37].

One of the strongest findings which emerged from this study was the significant decline in physical activity levels with age. Adults aged 75+ were over twice as likely to be inactive as those aged 60–64 years. A decline in physical activity during successive decades of life is common in cross-sectional [31,38] and prospective studies [17] from a range of countries in the developed world. The importance of age as a predicator of overall physical activity has also been demonstrated with objectively measured physical activity data [39]. Given the importance of regular physical activity to the maintenance of functional independence [40], considerable numbers of older Irish adults may be accelerating their functional decline through inactivity. There is evidence to suggest that embarking on a programme to comply with physical activity guidelines as late as 60 years of age may result in increasing life expectancy by an additional 1–2 years [41]. Therefore interventions which promote the adoption of physical activity in previously inactive older adults should be considered.

Adults with above secondary education were less likely to be classified as inactive than those who did not attend third level (OR 0.79 females, 0.89 males). This relationship has also been observed for secondary education, with Vagetti et al. [42] noting that older women who completed secondary education were 1.4 times more likely to meet physical activity guidelines that their peers with primary incomplete education. In previous research higher levels of education doubled the likelihood of participation in sports or exercise, however it was not associated with being active in walking or gardening [35]. Interestingly, Shaw and Spokane [38] found that education level moderated the association between age and physical activity such that education disparities in physical activity widened with increasing age. Collectively these studies demonstrate that different intervention strategies may be required depending on the education level of the target older adult population.

The importance of driving in maintaining independence, feelings of self-worth, and being connected to life and society has been noted previously [43]. In our study, older adults who did not own a car were more likely to be inactive than their peers who reported owning a car. This is consistent with findings from a cross-sectional survey of Scottish older adults which noted that those who did not have daily access to a car were more likely to be sedentary [44]. Research involving British older adults found that those with access to the car were consistently more likely to take part in more social activities [45]. Therefore older adults may use their car to get to places that permit them to be active.

Self-rated health is a reliable gauge of overall health status [46]. This has been demonstrated more recently in an older Irish population which found comorbidity to be a predictor of perceived health status [47]. In the present study older adults with poor perceptions of their health were more likely to be inactive than those who rated their health positively. Similar findings of a positive association between self-rated health and physical activity level have been observed in cross-sectional studies with older adults in Brazil, Italy and the US [31,42,48]. A positive perception of health status may be key to promoting and maintaining physical activity and quality of life in the elderly population [42]. However since this is a cross sectional study it is impossible to determine if physical activity leads to increased self-perceived health or if those with high self-perceived health do more physical activity [49].

Maintenance of physical function is important for independent living in older adults[50]. Those who are afraid of falling appear to enter a debilitating spiral of loss of confidence, restriction of physical activities and social participation, physical frailty, falls, and loss of independence [51]. In the present study adults who self-reported no fear of falling and no falls in the last year were less likely to be inactive. Given the cross-sectional nature of our data it is not possible to establish causality between physical activity and fear of falling, however it is encouraging to note that physical activity interventions have been effective in reducing fear of falling in community-living older people [51].

Proxy measures of social connectivity, such as attending a course or looking after grandchildren, were associated with physical activity level. There is some supporting evidence from a qualitative study where minority women noted that taking care of grandchildren kept them active [52]. Equally, older adults have also noted that being fit and able to play with grandchildren was one of the health benefits of physical activity [53]. Physical activity participation, particularly when performed with friends or in a group setting, can be an important contributor to social connectivity in this age-group.

Limitations of the study should be considered when interpreting findings. Firstly, the TILDA data only includes community-dwelling older adults, and therefore our findings cannot be generalised to those living in nursing homes or other institutions. Second, the exclusion of residents in long-stay institutions has led to an under-representation of the frailer older population in the TILDA dataset [12]. Third, there is a likelihood of misreporting of physical activity with IPAQ due to challenges in recall [26]. In addition this cross-sectional analysis precludes assessment of the nature and direction of the relationship between correlates and physical inactivity and therefore causality cannot be determined. Finally, the weighted analysis, which is recommended when using survey data to report on national populations, is likely to yield significant outcomes as the analysis is incorporating the population size rather than the sample size [54]. Strengths include the use of a large, population-representative sample and the extensive number of correlates available to assess in the context of physical inactivity. Future waves of TILDA will allow for longitudinal study and examination of determinants of physical activity in this Irish cohort.

### Conclusion

As Ireland, like many other developed countries, faces rapid population ageing, the promotion of regular engagement in physical activity is critical to sustain health and prevent disease among the older adult population. Our findings identify specific subgroups that are at particular risk of physical inactivity. In addition, this study demonstrates that gender strongly influences factors that affect physical activity participation among community-dwelling older adults living in Ireland. Future research should consider analysis of specific population subgroups, for example those with diabetes or with function-limiting conditions such as arthritis. Additionally, correlates research should consider other aspects of physical activity recommendations such as the performance of muscle strengthening and flexibility exercise. The results from this study can support the development of interventions to tackle inactivity in older adults and guide the creation of policy in Ireland which aims to promote healthy ageing.
